# Efficacy of futibatinib, an irreversible fibroblast growth factor receptor inhibitor, in *FGFR*-altered breast cancer

**DOI:** 10.1038/s41598-023-46586-y

**Published:** 2023-11-18

**Authors:** Turcin Saridogan, Argun Akcakanat, Ming Zhao, Kurt W. Evans, Erkan Yuca, Stephen Scott, Bryce P. Kirby, Xiaofeng Zheng, Min Jin Ha, Huiqin Chen, Patrick K. S. Ng, Timothy P. DiPeri, Gordon B. Mills, Jordi Rodon Ahnert, Senthil Damodaran, Funda Meric-Bernstam

**Affiliations:** 1 Department of Investigational Cancer Therapeutics, The University of Texas MD Anderson Cancer Center, 1400 Holcombe Boulevard, Unit 455, Houston, TX 77030 USA; 2 Department of Basic Oncology, Graduate School of Health Sciences, Hacettepe University, Ankara, 06100 Turkey; 3 Department of Bioinformatics and Computational Biology, The University of Texas MD Anderson Cancer Center, Houston, TX 77030 USA; 4 The Jackson Laboratory for Genomic Medicine, Farmington, CT 06032 USA; 5 Department of Pediatrics, University of Connecticut Health Center, Farmington, CT 06030 USA; 6 Department of Surgical Oncology, The University of Texas MD Anderson Cancer Center, Houston, TX 77030 USA; 7 Division of Oncological Sciences, Knight Cancer Institute, Oregon Health and Science University, Portland, OR 97239 USA; 8 Precision Oncology, Knight Cancer Institute, Oregon Health and Science University, Portland, OR 97239 USA; 9 Department of Breast Medical Oncology, The University of Texas MD Anderson Cancer Center, Houston, TX 77030 USA; 10 Department of Breast Surgical Oncology, The University of Texas MD Anderson Cancer Center, Houston, TX 77030 USA; 11 The Sheikh Khalifa Bin Zayed Al Nahyan Institute for Personalized Cancer Therapy, The University of Texas MD Anderson Cancer Center, Houston, TX 77030 USA; 12 Present Address: Department of Biostatistics, Graduate School of Public Health, Yonsei University, Seoul, Republic of Korea

**Keywords:** Breast cancer, Cancer therapy, Cancer genomics

## Abstract

Several alterations in fibroblast growth factor receptor (FGFR) genes have been found in breast cancer; however, they have not been well characterized as therapeutic targets. Futibatinib (TAS-120; Taiho) is a novel, selective, pan-FGFR inhibitor that inhibits FGFR1-4 at nanomolar concentrations. We sought to determine futibatinib’s efficacy in breast cancer models. Nine breast cancer patient–derived xenografts (PDXs) with various *FGFR1-4* alterations and expression levels were treated with futibatinib. Antitumor efficacy was evaluated by change in tumor volume and time to tumor doubling. Alterations indicating sensitization to futibatinib in vivo were further characterized in vitro*. FGFR* gene expression between patient tumors and matching PDXs was significantly correlated; however, overall PDXs had higher FGFR3-4 expression. Futibatinib inhibited tumor growth in 3 of 9 PDXs, with tumor stabilization in an *FGFR2*-amplified model and prolonged regression (> 110 days) in an FGFR2 Y375C mutant/amplified model. FGFR2 overexpression and, to a greater extent, FGFR2 Y375C expression in MCF10A cells enhanced cell growth and sensitivity to futibatinib. Per institutional and public databases, *FGFR2* mutations and amplifications had a population frequency of 1.1%–2.6% and 1.5%–2.5%, respectively, in breast cancer patients. *FGFR2* alterations in breast cancer may represent infrequent but highly promising targets for futibatinib.

## Introduction

Fibroblast growth factor receptor (FGFR) is a major member of the receptor tyrosine kinase family, which contains 4 classical FGFRs (FGFR1, FGFR2, FGFR3, and FGFR4). Genomic alterations in *FGFR*, are found in 343 (7.1%) of 4,853 solid tumors; the most commonly affected tumor types include breast cancer (18%), as well as urothelial (32%), endometrial (13%), and squamous lung (13%) cancers and intrahepatic cholangiocarcinoma (CCA; 3%–13%)^1^. Recently, FGFR was proven to be a compelling therapeutic target. The U.S. Food and Drug Administration (FDA) granted regulatory approvals for the use of the FGFR inhibitor erdafitinib in urothelial carcinoma with *FGFR2* or *FGFR3* fusions, or *FGFR3* mutations and the use of the FGFR inhibitors pemigatinib, infigratinib and most recently futibatinib in CCA with *FGFR* fusions or rearrangements^2–5^. FGFR inhibitors have reported activity in other tumor types, across a variety of alterations. However, currently there are no FGFR inhibitors with regulatory approval to treat breast cancer and further none approved for treatment of tumors with genomic amplification or RNA increases. Thus, there is a need to study biomarkers of sensitivity to FGFR inhibitors in breast cancer to facilitate clinical development.

*FGFR1* amplification, nuclear *FGFR1* expression, *FGFR3* overexpression, *FGFR4* overexpression, and *FGFR4* mutations are associated with endocrine therapy resistance in breast cancer^6–9^. *FGFR2* amplification is enriched in triple-negative breast cancer (TNBC) and occurs in 4% of patients with locally advanced/metastatic disease^10^. Taken together, these data suggest that *FGFR* alterations are important drivers in breast cancer and may represent a therapeutic opportunity.

Futibatinib (TAS-120; Taiho) is a novel, selective, pan-FGFR inhibitor that irreversibly binds to and inhibits FGFR1-4 at nanomolar concentrations^11^. In a multi-histology phase I trial, it had a manageable safety profile^12^. In the phase I expansion, 197 patients with advanced solid tumors were treated with the drug^13^. The objective response rate (ORR) was 13.7%, and responses were seen in patients with a broad spectrum of tumors, including CCA and breast, gastric, urothelial, central nervous system, and head and neck cancer^14^. Futibatinib demonstrated efficacy with durable responses in patients with intrahepatic CCA harboring *FGFR2* fusion/rearrangements in the pivotal FOENIX-CCA2 phase 2 study (NCT02052778). The confirmed ORR was 41.7% (43/103) and the disease control rate was 82.5%^15^; based on this data, the FDA granted accelerated approval to futibatinib for adult patients with previously treated, unresectable, locally advanced or metastatic intrahepatic CCA harboring *FGFR2* gene fusions or other rearrangements^5^. In this study, we aimed to elucidate the efficacy of futibatinib in breast cancer and identify the molecular features associated with its antitumor activity.

## Results

### *FGFR* alterations in breast cancer patient-derived xenograft models generated from primary or locoregionally recurrent tumors

**Figure 1:**
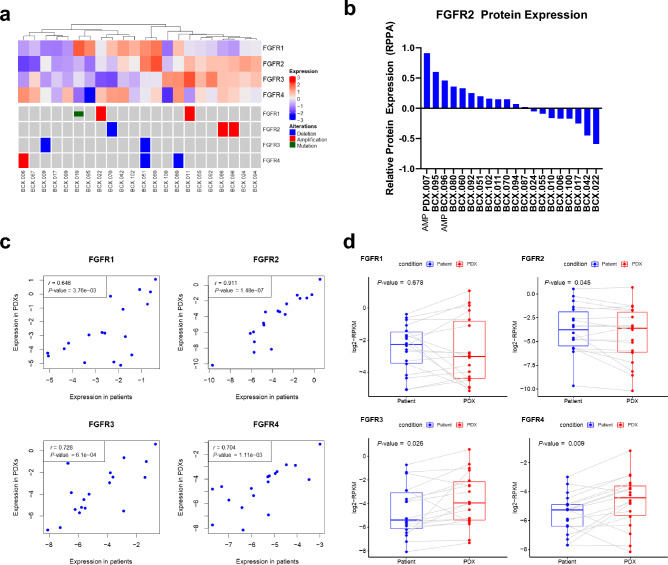
Fibroblast growth factor receptor (FGFR) alterations in breast cancer patient-derived xenografts (PDXs). (**a**) FGFR expression and genomic alterations from 22 PDXs generated from 21 patients**.** (**b**) Relative protein expression of breast cancer PDXs determined by reverse phase protein array (RPPA). (**c**) Relative FGFR1-4 mRNA expression in patient samples and matching PDX models. RNA expression is presented in log2-normalized reads per kilobase million (RPKM). A 2-sided paired Student *t* test was used to calculate *P*-values. (**d**) The correlation of expression between *FGFR* genes in patient samples and those in matching PDX models is shown. RNA expression is presented in log2-normalized RPKM. The Pearson correlation coefficient (*r*) was used to measure the statistical association between 2 variables.

We assessed the *FGFR* status in 21 breast cancer PDX models developed from primary or locoregionally recurrent tumors (Supplementary Table S1)^16^. These models demonstrated a variety of *FGFR* genomic alterations and varying FGFR RNA expression (Fig. 1A). Notably, BCX.066 and BCX.096 were developed from the same patient, from two metachronous local recurrences, with both PDXs demonstrating *FGFR2* amplification and similar FGFR expression patterns. The *FGFR2* amplification was also validated in the patient sample by in situ hybridization with clinical testing (data not shown). As FGFR-inhibitor sensitivity is also associated with FGFR RNA overexpression^17^, we also assessed FGFR1-4 RNA expression in the models. FGFR2 protein expression was determined for most of these breast PDXs as well as an additional *FGFR2* AMP PDX (PDX.007, described below) using reverse phase protein array (RPPA) (Fig. 1B).

Of these 21 models generated from neoadjuvant chemotherapy-resistant primary or recurrent tumors, we selected eight PDXs with a variety of FGFR DNA or RNA alterations for further study for the antitumor activity of futibatinib. The genomic characteristics of these models are described in Table 1, and the clinical characteristics of the patients are described in Supplementary Table S2. This included 2 models with *FGFR1* gene amplification, 1 with *FGFR2* amplification, and 1 with *FGFR4* amplification for testing. We also tested BCX.010, a model with high FGFR1 expression but a FGFR1 mutation V273M. This mutation is found in patients with Kallman syndrome and is thought to cause the loss of FGFR function^18,19^. We also selected 3 models with higher FGFR mRNA expression relative to the other models but without amplification: BCX.051 (FGFR1, FGFR2), BCX.095 (FGFR1), and BCX.080 (FGFR3 and FGFR4).

**Table 1:** *FGFR* alterations in selected patient-derived xenografts.

Model	Origin of biopsy	Subtype	FGFR mutations/amplifications	RNA relative overexpression
BCX.022	Primary	IDC	FGFR1 AMP	
BCX.011	Primary	IDC	FGFR1 AMP	
BCX.010	Primary	Metaplastic/ Sarcomatoid	FGFR1 V273M	
BCX.066	Locoregional recurrence	IDC	FGFR2 AMP	
BCX.006	Primary	IDC	FGFR4 AMP	
BCX.095	Primary	IDC		FGFR1
BCX.051	Primary	IDC		FGFR1/2
BCX.080	Primary	IDC		FGFR3/4
PDX.007	Distant metastasis (pleural fluid)	IDC	FGFR2 Y375C	
FGFR2 AMP	N/A*			

IDC, invasive ductal carcinoma; AMP, amplification; FGFR, fibroblast growth factor receptor**;** N/A, not applicable.

*RNAseq data not available.

Notably, 18 of the 21 PDXs with RNA sequencing (RNAseq) data also had matching patient tumors with RNAseq data. The FGFR gene expression in the patient tumors was significantly correlated with that in the PDXs: FGFR1, *r* = 0.646; FGFR2, *r* = 0.911; FGFR3, *r* = 0.728; and FGFR4, *r* = 0.704 (Fig. 1C). However, FGFR3 (*P* = 0.026) and FGFR4 (*P* = 0.009) showed significantly higher expression in the PDXs than in the patient tumors at the population level (Fig. 1D). Because the correlation of the FGFR gene expression between the patient tumors and PDXs were moderate to strong, we did not determine whether the change in expression was related to the replacement of human stromal genes by mouse stromal genes that are not detected with human RNAseq probes or whether there was true FGFR upregulation in some models’ cancer cells. The *FGFR2*- amplified models showed relatively high mRNA expression of the respective genes, while the *FGFR1*-amplified models showed only low-to-moderate levels of mRNA expression of FGFR1 (Supplementary Fig. S1).

### Development of MDA-PDX.007, a *FGFR2* amplified/mutant PDX model

In addition, we developed a new *FGFR*-altered PDX model, PDX.007, from the pleural fluid of a patient with metastatic breast cancer. PDX.007 was obtained from a 55-year-old white woman who had very aggressive TNBC with invasive ductal histology. She received neoadjuvant therapy with paclitaxel followed by doxorubicin and cyclophosphamide. The patient underwent surgery and radiation therapy, and developed a locoregional relapse within a month of completing radiation and distant recurrence approximately 3 months after that. She was treated with a combination of carboplatin and gemcitabine, and subsequently with ixabepilone and capecitabine, followed by an investigational targeted therapy/immunotherapy combination, but her cancer progressed rapidly. Genomic profiling with an Ampliseq 50-Gene Somatic Mutation Analysis Panel, which detects hot spot mutations, showed that the patient had the FGFR2 Y375C somatic missense mutation. PDX.007 was generated from pleural fluid collected after the patient’s cancer progressed despite the treatments described above. The PDX was characterized with estrogen receptor, progesterone receptor, and human epidermal growth factor 2 immunohistochemical analysis, which confirmed that the PDX was TNBC. Whole-exome sequencing of the PDX demonstrated FGFR2 Y375C mutation and *FGFR2* amplification. This model also demonstrated high FGFR2 protein expression on RPPA.

### Antitumor activity of futibatinib in *FGFR*-altered breast cancer PDX models

**Figure 2:**
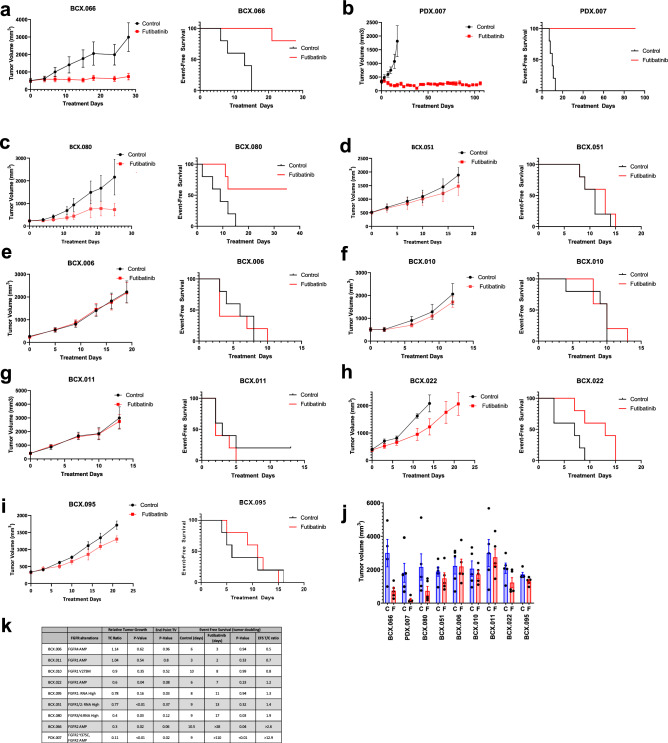
Response of patient-derived xenografts (PDXs) to the fibroblast growth factor receptor (FGFR) inhibitor futibatinib. (**a**–**i**) Female mice bearing breast cancer PDXs (n = 5 per group) were treated orally with the vehicle control or futibatinib (15 mg/kg/day). The treatment was stopped at 28 days or when the tumor diameter reached 1.5 cm^3^, except for mice bearing the PDX.007 model, in which the treatment was stopped on day 110. Growth (left) and event-free survival duration (right, defined as days until tumor doubling) curves are shown for each model. Growth curve graphs show the mean change in TV (mm^3^) ± the standard errors of the means. (**j**) Tumor volume of mice in control and futibatinib groups were measured at the last day of vehicle treatment. Bars, error bars, and dots show mean tumor volume (mm^3^), SEM, and individual tumor volume of each mouse, respectively. C, control; F, futibatinib. (**k**) Relative tumor growth tumor volume treatment/control ratios (TV T/C ratio) were calculated on day 21. End point tumor volumes (TV) were calculated at the last day of vehicle treatment. Event free survival (EFS) was calculated at the last day of futibatinib treatment. *This model does not have RNAseq data.

We tested the antitumor activity of futibatinib in 9 PDX models: eight PDX models with FGFR alterations generated from surgical samples described above, and in the one *FGFR2* amplified/ FGFR2 Y375C mutated model derived from a patient with metastatic breast cancer. These nine PDX models were treated with oral futibatinib (15 mg/kg daily), and body weights and tumor volumes were monitored. The drug did not promote weight loss in any of the models (data not shown). Antitumor efficacy was assessed by assessing change in tumor volume (TV), using the TV treatment/control (T/C) ratio (Supplementary Fig. S2) and event-free survival (EFS). Significant anti-tumor efficacy was observed in 2 models: BCX.066 (Fig. 2A), which had *FGFR2* amplification/overexpression; and PDX.007 (Fig. 2B), which had FGFR2 Y375C mutation and *FGFR2* amplification. More limited antitumor effects were seen in BCX.080, which had high RNA expression of FGFR3 and FGFR4 (Fig. 2C). At day 21, BCX.080, BCX.066, and PDX.007 had TV T/C ratios of 0.40, 0.30, and 0.10, respectively. The Kaplan–Meier curves assessing time to tumor doubling demonstrated that these three models showed statistically significant improvements in EFS durations with futibatinib treatment (Fig. 2A–C,K). Futibatinib treatment more than doubled the EFS of BCX.066 and PDX.007. Futibatinib treatment caused PDX.007 to regress from baseline (best response: mean − 72%), and disease control was maintained for more than 100 days. The median EFS durations of the control and futibatinib groups for PDX.007 were 9 days and more than 110 days, respectively (*P* = 0.002; EFS T/C ratio > 12.9). The remaining 6 models did not show an improvement in EFS durations (Fig. 2D–I,K).

Tumor volumes of control and futibatinib treatment groups were compared on the last day of vehicle treatment (Fig. 2J). Futibatinib treatment had the most significant antitumor activity in PDX.007 (*P* = 0.023) and BCX.066 (*P* = 0.020) models (Fig. 2K). FGFR status, relative tumor growth, end point tumor volume, and EFS calculations were summarized in Fig. 2K.

### FGFR2 Y375C enhances cell proliferation, FGFR signaling, and sensitivity to FGFR inhibitors in vitro

**Figure 3:**
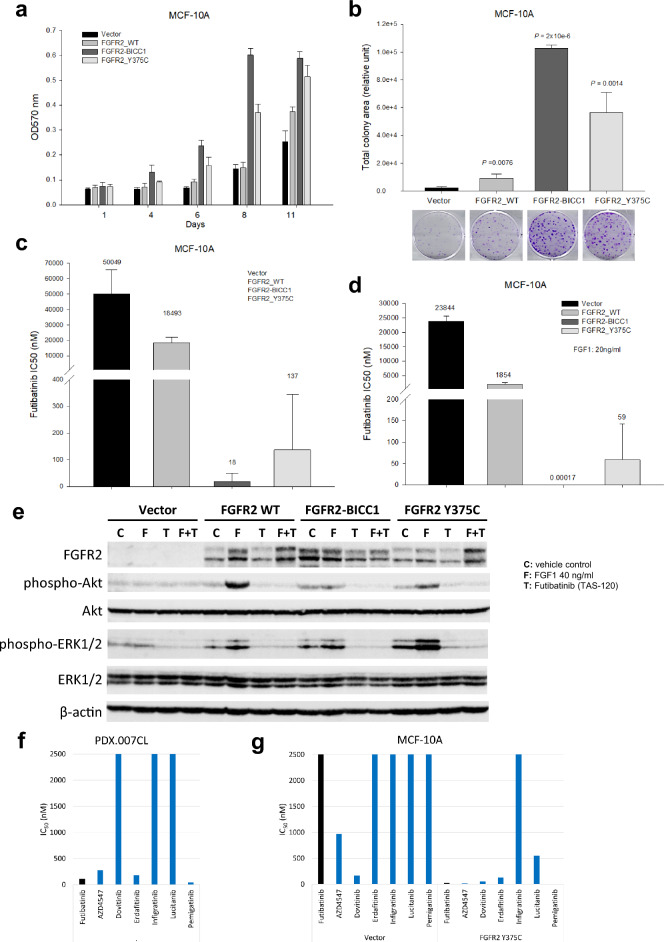
Effects of *FGFR2* alterations on cell sensitivity to fibroblast growth factor receptor (FGFR) inhibitors and FGFR signaling. Normal mammary epithelial MCF10A cells were transduced with *FGFR2-BICC1* fusion, FGFR2 Y375C mutation, *FGFR2* wild type (WT), and the vector control. (**a**) Cell viability was measured by a sulforhodamine B (SRB) assay. Bars show the mean OD570 ± the standard errors of the means. (**b**) Cells were cultured for 3 weeks. Cell colonies were stained, and the total colony area was quantitated. The comparisons of the total colony areas of the *FGFR2* WT, *FGFR2-BICC1*, and FGFR2 Y375C cell lines to the vector control cell line. Bars show the mean total colony areas ± the standard errors of the means. Below, representative stained plates are shown. (**c**) Cells were and treated with a serial dilution of futibatinib for 4 days. Cell viability was assessed with an SRB assay, and the half-maximal inhibitory concentration (IC_50_) values were calculated. Bars show the mean IC_50_ values ± the standard errors of the means. (**d**) While being starved, cells were treated with fibroblast growth factor (FGF1) for 24 h and various doses of futibatinib for 4 days. Cell viability was assessed with an SRB assay, and IC_50_ values were calculated. Bars show mean IC_50_ values ± the standard errors of the means. (**e**) Cells were starved for 24 h and treated with futibatinib at 0.2 µM for 3 h 45 min, followed by FGF1 for 15 min. Immunoblotting was performed using antibodies against FGFR2, p-Akt (S473), Akt, p-ERK1/2 (T202/Y204), ERK1/2, and β-actin. (**f**) PDX.007CL cells were plated in spheroid plates and treated a panel of FGFR inhibitors for five days. A luminescence assay was used to determine cell viability and IC_50_ values were calculated. (**g**) MCF10A vector control and FGFR2 Y375C cells were treated with a panel of FGFR inhibitors for four days. Sulforhodamine B assay was used to determine cell viability and IC_50_ values were calculated.

Given the robust effect of futibatinib on the PDX.007 model with FGFR2 Y375C mutation as well as *FGFR2* amplification, to try to dissect the contribution of *FGFR2* mutation on oncogenicity and futibatinib sensitivity, we analyzed the impact of the FGFR2 Y375C mutation and FGFR2 overexpression on the breast epithelial cell line MCF10A using stable viral transduction^20^. Although MCF10A is a normal-like cell line that differs from cancer cells in many ways, expression of potential oncogenic drivers in MCF10A line has often been used as a functional genomic screen in prior studies^20,21^. As *FGFR* fusions are established oncogenic drivers, *FGFR2-BICC1* fusion was used a control. Time-course experiments showed that cell growth was substantially increased in cells with the FGFR2 Y375C mutation compared with vector-control and wild-type (WT) *FGFR2* cells (Fig. 3A). Next, we performed a colony formation assay with the same cell lines. The results showed that overexpression of the FGFR2 Y375C mutation enhanced colony formation to a greater extent than did *FGFR2* WT overexpression. Although not as robust as the increase seen with the FGFR2 Y375C mutation, *FGFR2* WT overexpression also led to a statistically significant increase in colony formation compared to vector control (*P* = 0.0076) (Fig. 3B).

Next, we assessed the impact of these alterations on cells’ sensitivity to futibatinib. FGFR2 overexpression reduced the IC_50_ from 50,049 nM to 18,493 nM. The FGFR2 Y375C mutation and FGFR2-BICC1 fusion reduced the futibatinib IC_50_ more dramatically, from 50,049 nM to 137 nM and 18 nM, respectively (Fig. 3C). Adding FGF1, a multifunctional ligand protein that binds and stimulates FGFR signaling, increased the sensitivity of all the cell lines. The pattern of sensitivity was similar to that seen in the absence of FGF1, with the exception that the addition of FGF1 ligand significantly increased futibatinib sensitivity in the FGFR2 WT cell line compared with the vector control (Fig. 3D) but had less of an effect on the sensitivity of the FGFR2 mutant or fusion cell lines. This is consistent with a model where the FGFR2 mutant and fusion construct are active in the absence of exogenous ligand. To confirm these results in another cell line, cholangiocycte cell line H69, was transduced using the same constructs. FGFR2 Y375C cells showed increased cell growth in a time course, colony formation, and sensitivity to futibatinib (Supplementary Fig. S3).

To investigate the molecular mechanisms underlying the driver effect of the FGFR2 Y375C mutation on cell growth and sensitivity to futibatinib, we used immunoblotting to evaluate FGFR downstream signaling in the cell lines. In contrast to the vector-control MCF10A cells, the other 3 stably transduced cell lines expressed FGFR2 proteins as expected (Fig. 3E). Following cell starvation, we treated the cells with FGF1, futibatinib, or futibatinib followed by FGF1. Compared to control cells, cells overexpressing WT and mutant FGFR2 proteins had increased basal levels of ERK1/2 phosphorylation, again consistent with constitutive activation. Cells with FGFR2-BICC1 and FGFR2 Y375C alterations also had slightly increased basal levels of phospho-Akt (p-Akt). These changes were further enhanced upon FGF ligand treatment, with the most dramatic effects being increased p-Akt levels in the FGFR WT cell line and increased p-ERK1/2 levels in the Y375C cell line. In all cell lines, futibatinib at 0.2 µM abolished the phosphorylation of both Akt and ERK1/2, which was not restored by FGF1 administration following futibatinib treatment. Interestingly, we noticed that futibatinib not only decreased FGFR signaling but also decreased FGFR2 protein expression in these cells (Fig. 3E).

We generated a cell line, PDX.007CL, from PDX.007 that bears the FGFR2 Y375C mutation and amplification. In addition to futibatinib, we tested the antitumor activity of six additional FGFR inhibitors (AZD4547, dovitinib, erdafitinib, infigratinib, lucitanib and pemigatinib) and compared inhibitory efficacy of these agents with futibatinib. The cells had varying sensitivity to different agents but were particularly sensitive to futibatinib and pemigatinib (Fig. 3F). We also compared these agents using MCF10A cells, FGFR2 Y375C mutation sensitized cells to all FGFR inhibitors, particularly to futibatinib, AZD4547 and pemigatinib (Fig. 3G). Exact IC_50_ values of PDX.007CL cell and all MCF10A FGFR2 constructs were presented in Supplementary Table S3. Growth inhibitory curves were presented in Supplementary Fig. S4.

### *FGFR* mutations and *FGFR2* amplification in breast cancer

**Figure 4:**
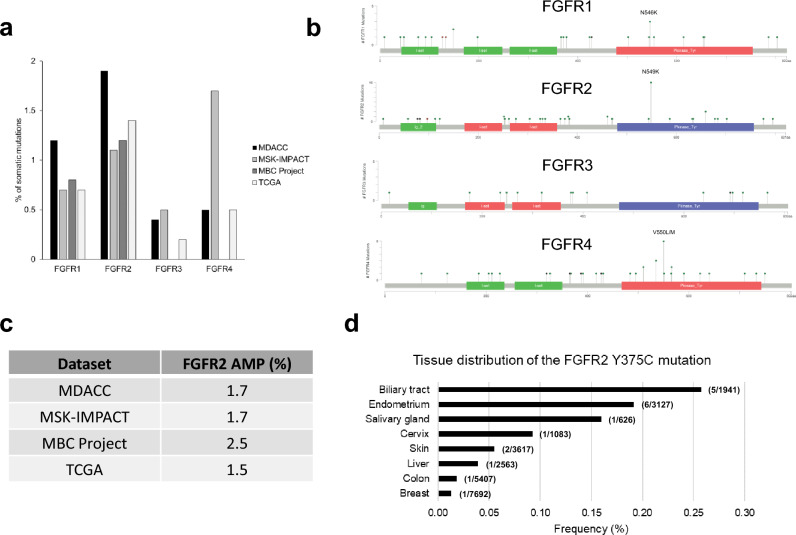
*FGFR* mutations in patients with breast cancer. Breast cancer data in MD Anderson Cancer Center (MDACC), MSK-IMPACT, Metastatic Breast Cancer (MBC) Project, and The Cancer Genomics Project (TCGA) databases were downloaded and compiled. (**a**) The bars show the percentages of somatic *FGFR* mutations in patients with breast cancer in each of these databases. (**b**) The 4 databases were analyzed together, and all mutations on the individual genes were illustrated using the cBioPortal MutationMapper tool. (**c**) Percentages of samples with *FGFR2* amplification (AMP) in each database. (**d**) Distribution of FGFR2 Y375C mutations in different tissues in the Catalogue of Somatic Mutations in Cancer (COSMIC) database.

In breast cancer, *FGFR* genes are frequently amplified or overexpressed but are not commonly mutated. To determine how often *FGFR* mutations are encountered, we downloaded and compiled breast cancer *FGFR* alteration data from the MD Anderson Institute of Personalized Cancer Therapy database, with data on *FGFR* mutations and copy number changes in 2635 and 1374 breast cancer patients respectively. In addition, we used data from the cBioPortal, an open access repository, to access the frequency of *FGFR* mutations in the MSK-IMPACT Clinical Sequencing Cohort (n = 1324), Metastatic Breast Cancer (MBC) Project (n = 237), and The Cancer Genome Atlas (TCGA) breast cancer data (n = 1098)^22–25^. *FGFR2* mutations were the most frequent (1.1% to 2.6%), and *FGFR3* mutations were the least frequent (Fig. 4A). The most common mutations were N549K for FGFR2, V550L/M for FGFR4, and N546K for FGFR1 (Fig. 4B). *FGFR2* amplification was also found at a prevalence of 1.5% to 2.5% (Fig. 4C).

### FGFR2 Y375C mutation in other tumor types

Next, we searched the Catalogue of Somatic Mutations in Cancer (COSMIC) database to find out whether the FGFR2 Y375C mutation occurs in other tumor types^26^. Overall, as expected, the frequency of the FGFR2 Y375C mutation was low; it was less than 0.3% across all of the histologies inspected. However, interestingly, we found that several non–breast cancer tissues had higher frequencies of FGFR2 Y375C expression than breast cancer tissues did. The highest frequency observed, 0.26%, was in biliary tract tumors, a tumor type known to be driven by *FGFR2* alterations (Fig. 4D). The other tumors where we encountered this mutation were, in order of decreasing mutation frequency, endometrium, salivary gland, cervix, skin, liver, colon, and, finally, breast. In the COSMIC database, the frequency of the FGFR2 Y375C mutation in breast cancer was 0.01% (Fig. 4D).

## Discussion

FGFR signaling has been shown to be a cancer driver. It is also a proven therapeutic target; the FDA has approved the use of pemigatinib, infigratinib and futibatinib for cholangiocarcinoma with *FGFR2* fusions and erdafatinib for bladder cancer with *FGFR2/3* fusions and *FGFR3* mutations*.* Several FGFR inhibitors, such as infigratinib, erdafitinib, AZD4547, and dovitinib, have been evaluated preclinically in breast cancer^27–30^. Still, to date, there are no FGFR inhibitors approved by the FDA to treat breast cancer. Thus, there is a need to study biomarkers of sensitivity to FGFR inhibitors in breast cancer to facilitate clinical development. We tested the antitumor activity of futibatinib, a novel, selective, pan-FGFR inhibitor, in 9 breast cancer PDX models with different molecular backgrounds. The results suggest that our models with an *FGFR2*-activating mutation and *FGFR2* amplification were sensitive to futibatinib. Further, we showed that the FGFR2 Y375C mutation enhances cancer growth and is targetable by the FGFR inhibitor futibatinib.

FGFR is frequently amplified in breast cancer and has been associated with poor prognosis and resistance to endocrine therapy and CDK4/6 inhibitors^6–9,31–38^. Many preclinical studies investigating FGFR in breast cancer have been conducted, mainly with selected breast cancer cell lines and cell line–derived xenografts^39–41^. However, to date, preclinical data have not effectively been translated into clinical activity. For example, dovitinib, a multi-tyrosine kinase inhibitor that inhibits FGFR1-3, vascular endothelial growth factor receptors 1–3, and platelet-derived growth factor receptor, preferentially inhibited the growth of *FGFR1*- and *FGFR2*-amplified cell lines in vitro and inhibited the growth of *FGFR1*-amplified xenografts^29^. However, in a phase II trial of dovitinib, there were no confirmed, objective responses^29^. A phase II randomized trial of fulvestrant with dovitinib vs. placebo showed that compared to patients without FGFR pathway alterations, those with FGFR pathway alterations had a higher objective response rate (27.7% vs. 10%) and longer progression-free survival duration (10.9 months vs. 5.5 months)^42^. Although the risk reduction reached significance only in the FGFR-altered group, a similar risk reduction was observed in patients without FGFR alterations. Thus, the degree to which the results were attributable to FGFR inhibition is unclear, as dovitinib is not a selective FGFR inhibitor. 38.3% of patients in the dovitinib arm discontinued therapy because of adverse effects. Lucitanib, another multikinase inhibitor with FGFR-inhibitory activity, was tested in a phase II trial in patients with metastatic breast cancer^43^. Although the prespecified efficacy target was met with an objective response rate of 19% in patients with *FGFR1*-amplified breast cancer, there was significant hypertension-related toxicity, limiting further development of the drug. Long-term tolerability issues were also reported with the combination of nintedanib and letrozole^44^. Several other multikinase inhibitors, as well as selective FGFR inhibitors, are in clinical trials as monotherapies and combination therapies for breast cancer (e.g., NCT04504331; NCT04936295, NCT03238196; NCT04742959; NCT04024436).

There has been great interest in using gene amplification to select patients for therapy with FGFR inhibitors. However, the frequency of *FGFR* amplification in different series has varied considerably, likely because of the different assays used (e.g., fluorescence in situ hybridization vs. Next-Generation Sequencing [NGS])^28^. On comprehensive genomic profiling, about 7.1% of cancer patients have *FGFR* alterations, most of which (66%) are amplifications^1^. However, there were no objective responses to FGFR inhibitor AZD4547 in the tumor-agnostic NCI-MATCH study, nor were there any patients with a progression-free survival duration of 6 months or longer among the 17 patients with *FGFR1* amplification and the 3 with *FGFR2* amplification treated with AZD4547^45^. Non–NGS-based patient selection strategies have been more challenging, as they require central screening. In a different translational study of AZD4547, 341 patients were screened with fluorescence in situ hybridization; *FGFR1* amplification was found in 18% of advanced HER2-negative breast cancers and 9% of advanced gastroesophageal cancers^46^. Notably, 1 (12.5%) of 8 breast cancer patients with *FGFR1* amplification and 3 (33.3%) of 9 gastroesophageal cancer patients with *FGFR2* amplification had confirmed responses with AZD4547. Preclinical studies demonstrated that high-level clonal amplification of *FGFR2* activates a distinct phenotype, bringing phosphoinositide 3-kinase/mTOR signaling under the control of FGFR2 and predicting sensitivity to FGFR inhibition^46^. These data, along with the growth inhibition we observed in the *FGFR2*-amplified breast cancer models in our study, support the need to further explore the efficacy of FGFR inhibitors against tumors with high-level, clonal *FGFR2* amplifications.

There has also been interest in using RNA expression for patient selection. In preclinical studies, the efficacy of the FGFR inhibitor rogaratinib was strongly correlated with RNA expression^47^. Sanchez-Guixe et al. tested the efficacy of rogaratinib in a series of breast cancer PDXs and reported that a composite score based on FGFR1-4 RNA expression was correlated with the drug’s antitumor efficacy^48^. In our study, *FGFR* amplification and overexpression were not concordant in many models. Although we do not have insight into mechanism of this discordance, lack of concordance between FGFR copy number status and expression had previously been reported in other studies^49,50^. The *FGFR2*-amplified BCX.066 model, whose growth was significantly inhibited by futibatinib, also demonstrated overexpression of FGFR2 RNA. In BCX.080, a model with FGFR3 and FGFR4 RNA overexpression and without a detectable genomic alteration, futibatinib had modest tumor growth inhibition without significantly prolonging EFS. As RNA-based assays are increasingly used to evaluate cancer patients, further work is needed to determine the predictive value of FGFR RNA expression. Notably PDX007 and BCX.096 (derived from the same patient as BCX066) had higher FGFR2 expression at the protein level by RPPA, also raising a potential role for FGFR2 protein expression testing.

Here, we demonstrated that FGFR2 Y375C, in the juxtamembrane domain of FGFR2 (exon 11), is an activating mutation that enhances the growth of MCF10A normal-like breast cells. Y375C mutations were previously described as heterozygous germline mutations in newborns with caudal appendages and craniosynostosis and have been postulated to activate these conditions^51–55^. Although Y375C mutations are uncommon, they have been reported in multiple tumor types^1,26,56,57^. A similar site, FGFR3 Y373C aligns with FGFR2 Y375C and is also known to be an activating mutation that is sensitive to FGFR inhibitors in preclinical models^1^. We also demonstrated that the FGFR2 Y375C mutation, like the *FGFR2-BICC1* fusion, is activating in model systems and confers sensitivity to futibatinib. This finding suggests that, although most drug development for FGFR to date has focused on fusions and amplifications, activating *FGFR* mutations may indeed predict FGFR-inhibitor sensitivity in breast cancer, as has been shown in bladder cancer. Although activating *FGFR2* mutations are not very common, several activating *FGFR2* mutations other than FGFR2 Y375C have been described in breast cancer^58^. Further study is needed to determine which FGFR alterations are activating and responsive to futibatinib or other FGFR inhibitors. *FGFR2* mutations have been reported in 10% to 12% of endometrial cancers and are associated with shorter disease-free and overall survival durations, suggesting another therapeutic opportunity^57,59,60^.

FGFR inhibitors may differ in their activity against different *FGFR* mutations. Goyal et al. reported that FGFR inhibitors have distinct activity profiles against secondary FGFR2 kinase mutations in intrahepatic cholangiocarcinoma cell lines, and that futibatinib was active against multiple *FGFR2* mutations conferring resistance to infigratinib or Debio 1347^61^. We also show that antitumor activity of different FGFR-targeting agents may vary in the FGFR2 Y375C bearing PDX.007CL and MCF10A transfected cell lines, highlighting potential need to match specific mutations with effective FGFR inhibitors in precision oncology.

Notably, we tested 2 models with *FGFR* mutations. One was highly sensitive to futibatinib, and one was not. The FGFR1 mutation V273M in the BCX.010 model is not thought to be a gain-of-function mutation^18,19^. This mutation is found in patients with Kallman syndrome, and structural modeling predicts that this mutation and others found in patients with the condition result in a loss of function. The methionine residue has a bulkier side chain than valine and is thought to structurally destabilize the D3 region in which it resides^18,19^. Consistent with this theory, our findings did not show significant antitumor efficacy in the BCX.010 model. In contrast, futibatinib led to durable growth inhibition in PDX.007 in the context of an activating *FGFR2* mutation. Unfortunately, this alteration was detected too late in this patient’s treatment course to allow treatment with an FGFR inhibitor. These 2 examples highlight the importance of pursuing comprehensive genomic characterization early in the treatment course of tumors with aggressive biology, as well as the need for functional annotation of genomic alterations, as not all genomic alterations are equally actionable.

Our study had some limitations. Although the paper presents multiple new PDXs, ultimately we are testing a limited number of models, and further study is needed, especially in ER + models with and without combination therapies. We tested the effect of *FGFR2* amplification and mutation in the MCF10A cell line, a normal-like cell line. Although this is a commonly used model for such assays, it is an EGF-dependent cell line, which complicates interpretation. Notably, we have also demonstrated the effect of this mutation when introduced in H69, a biliary cell line, however, testing its effect in other breast cancer cell line backgrounds may have value. Testing effect of other non-activating FGFR2 SNVs, as well as effect of introducing the mutation with CRISPR may provide additional controls. We did limited number of experiments to look at the effect of futibatinib on cell signaling in vitro. Further study is needed to assess the pharmacodynamic effects of futibatinib in patients and the determine target inhibition as well as adaptive responses in different genomic backgrounds.

In the phase I expansion trial of futibatinib across all diseases, although responses were most common among patients with *FGFR* fusions, 6 patients who had a partial response to futibatinib treatment had activating *FGFR* mutations: 1 had a mutation in *FGFR1*, 3 had a mutation in *FGFR2,* and 2 had a mutation in *FGFR3*^14^. Importantly, a confirmed response was observed in a patient with an unknown primary tumor bearing the FGFR2 Y375C mutation. This patient had a progression-free survival duration of 19.2 months and a response duration of 10.3 months. Further, a patient with *FGFR2*-amplified TNBC responded to futibatinib and had a progression-free survival duration of 22.1 months and a response duration of 20.8 months. Interestingly, FGFR inhibitor activity was recently noted in patients with FGFR2 Y375C mutations in two additional reports. In RAGNAR, the Phase II study of erdafatinib in patients with solid tumors with FGFR alterations reported by Pant et al.^62^, 73 patients were enrolled with *FGFR* mutations, of whom, 25 had a FGFR2 Y375C mutation (per supplementary data). Although the response rate in the patients with FGFR2 Y375C was not specifically reported, one of the complete responders had salivary cancer with a FGFR2 Y375C mutation. Further, in a recent report of the selective FGFR2 inhibitor RLY-4008, Subbiah et al. reported that a patient with a salivary tumor bearing FGFR2 Y375C had a prolonged partial response with substantial symptomatic improvement^63^.

In our study we saw significant growth inhibition in a PDX model with FGFR2 amplification and tumor regression in a model with *FGFR2* amplification and FGFR2 Y375C mutation. This suggests FGFR2 amplification *may* sensitize to FGFR inhibition. Although it is hard to dissect the attribution of futibatinib sensitivity of PDX007 s to FGFR2 amplification vs mutation, our in vitro data demonstrates that FGFR2 Y375C is oncogenic and sensitive to FGFR inhibition. This data is now supported by recent clinical trial reports with three different FGFR inhibitors, demonstrating objective responses in patients with FGFR2 Y375C mutations. Thus, FGFR inhibitor therapy should be considered for patients bearing these alterations. Notably, the ASCO TAPUR trial is currently testing the antitumor efficacy of futibatinib in solid tumors with FGFR mutations or fusions.

## Conclusions

We report the antitumor activity of futibatinib, a selective, irreversible pan-FGFR inhibitor, in both in vivo and in vitro settings. The major strength of this study was its analysis of futibatinib response in multiple breast cancer PDXs with diverse molecular alterations. Notably although the models tested had a variety of FGFR alterations, most did not appear to be driven by FGFR signaling. Futibatinib treatment led to significant growth inhibition in models with *FGFR2* amplification, further supporting the rationale for this alteration being pursued in a phase II breast cancer trial (NCT04024436). We also demonstrated that the FGFR2 Y375C mutation is activating and sensitizes cells to futibatinib treatment in vitro and that futibatinib treatment in an FGFR2 Y375C–bearing PDX led to durable regression in vivo. These findings suggest that *FGFR2*-activating mutations are therapeutic targets and further study is needed in FGFR inhibitors in tumors driven by activating *FGFR* mutations and *FGFR2* amplification. Further, our data suggest that PDX-based screening can identify clinically relevant predictive markers for emerging therapies.

## Methods

### Patient-derived xenograft models and molecular characterization

All experiments on humans/human samples were approved by the MD Anderson Cancer Center (MDACC) Institutional Review Board. All methods involving human studies were carried out in accordance with the ethics principles of the Declaration of Helsinki and the International Council of Harmonization Guidelines on Good Clinical Practice. A written informed consent was obtained from all participating patients. All animal experiments were approved by MDACC’s Animal Care and Use Committee (protocol #00,001,405-RN01), which is accredited by the Association for Assessment and Accreditation of Laboratory Animal Care (AAALAC). All animal experiments were performed in accordance with the approved protocol, in accordance with the Institutional Animal Care and Use Committee (IACUC) guidelines. Animal experiments were done, analyzed, and presented in accordance with Animal research: Reporting i*n vivo* experiments (ARRIVE) guidelines.

Patient-derived xenografts (PDXs) were established by implanting tumor fragments or pleural fluid cell pellets into the flanks of female nude mice (Envigo/Harlan Labs) or NOD.Cg-Prkdcscid Il2rgtm1Wjl/SzJ mice (Jackson Laboratory) as described previously^64^. All animal experiments were approved by MD Anderson’s Institutional Animal Care and Use Committee. Genomic DNA was isolated from frozen or formalin-fixed, paraffin-embedded tumor tissue using the QIAamp DNA Mini Kit (Qiagen). Short tandem repeat DNA fingerprinting was performed with the AmpFLSTR Identifiler kit (Applied Biosystems) at MD Anderson’s Cytogenetics and Cell Authentication Core Facility. DNA sequencing and RNA sequencing were performed as described previously^16^. PDX models were sequenced on either a targeted exome platform (T200, Supplementary Table S4) or by whole exome sequencing.

### In vivo treatment

Futibatinib was dissolved in 0.5% hydroxypropyl methylcellulose (Sigma-Aldrich) in distilled water. Female athymic nude mice were randomized to receive 15 mg/kg futibatinib or the vehicle control by oral gavage daily. Body weights were monitored, and the perpendicular diameters of each tumor were measured twice a week with a digital caliper. Treatment testing was performed using subcutaneous implantation.

### Cell lines and culture

The normal-like mammary epithelial cell line MCF10A was obtained from the American Tissue Culture Collection. The normal human cholangiocyte cell line H69 was a gift from Dr. Gregory Gores’ laboratory at Mayo Clinic, Rochester, Minnesota. Both cell lines were verified to be free of mycoplasma contamination. MCF10A cells were cultured in complete mammary epithelial cell growth medium (MEGM) supplemented with growth factors and insulin (Lonza) as well as 10% fetal bovine serum (FBS). For cell starvation, MCF10A cells were cultured in MEGM medium without the supplemented materials and FBS. H69 cells were cultured in RPMI-1640 supplemented with 10% FBS.

PDX.007CL cell line was derived from PDX.007 xenograft model. Briefly, xenograft tumor was digested and filtered through a 100 μm cell strainer. Cells were cultured in DMEM/F12 medium supplemented with 5% FBS and kept in ultra-low attachment flasks for two months. Later cells were transferred to regular culture flasks and authenticated using short tandem repeat analysis.

### Viral transfection, drugs, and other in vitro reagents

Lentiviral constructs expressing *FGFR2* WT, the *FGFR2-BICC1* fusion, or the FGFR2 Y375C mutation were created as described previously^20^. MCF10A and H69 cells were infected with virus packaged from HEK 293 T cells using viral expression vectors as well as an empty vector (#PS100064) (OriGene). Futibatinib was provided by Taiho Oncology Inc. AZD4547 and erdafitinib were purchased from Medkoo Biosciences, Inc. Dovitinib, infigratinib and lucitanib were purchased from Selleck Chemicals. Pemigatinib (NSC 816,556) was obtained from the National Cancer Institute, Division of Cancer Treatment and Diagnosis, Developmental Therapeutics Program. For in vitro experiments, stock solutions of all drugs were prepared in DMSO (Sigma-Aldrich). FGF1 was purchased from R&D Systems. Immunoblotting antibodies, including anti-FGFR2 (#11,835), anti–phospho-ERK1/2 (T202/Y204; #4370), and anti–phospho-Akt (S473; #4060), were purchased from Cell Signaling Technology. Anti–β-actin antibody (#A5441) was purchased from Sigma. The secondary antibodies goat-anti-rabbit Alexa Fluor 680 (#A21076) and goat-anti-mouse Dylight 800 (#610,145–121) were purchased from Life Technologies and Rockland Immunochemicals, respectively.

### Western blot analysis

Cells were washed with cold phosphate-buffered saline and lysed in 2 × Laemmli buffer. Protein was quantified using a Pierce BCA Protein Assay Kit (ThermoFisher) before being loaded onto the gel. After sodium dodecyl sulphate–polyacrylamide gel electrophoresis, the protein was transferred to a 0.2-μm nitrocellulose membrane (Bio-Rad Laboratories). Membranes were blocked with 0.1% casein blocking buffer. Immunoblotting was performed with the primary antibodies at room temperature overnight, followed by washing and probing with secondary antibodies with fluorescence conjugation. The immunoblots were visualized using the Odyssey IR Imaging System (Li-Cor). Image Studio software, v4.0, was used to analyze the bands.

### Cell proliferation assays

MCF10A and H69 cells were seeded in 96-well plates at densities of 3000 to 6000 cells per well, depending on the growth characteristics of each cell line. After cells adhered overnight, titrating concentrations of futibatinib were added to the wells in triplicate, and the cells were incubated at 37 °C for 4 days. Cell viability was measured using a sulforhodamine B (SRB) assay. Optical density values were read at 570 nm (Synergy 4, BioTek). The half-maximal inhibitory concentration (IC_50_) was using CalcuSyn software (Biosoft).

For multiple drug comparisons, PDX.007CL cells were counted and plated in 384-well spheroid plates (Corning Inc.). Five days later spheroids were confirmed under the microscope, and they were treated with serial dilutions of the drugs in quadruplicates. Five days later cell viability was measured using a luminescence-based assay (CellTiter Glo 3D, Promega Corporation). MCF10A cells were counted and plated in 384-well solid white plates. The following day cells were treated with serial dilutions of the drugs in quadruplicates. Four days later cell viability was measured using a luminescence-based assay. The IC_50_ values were determined using CalcuSyn.

### Colony formation assay

Cells were plated in triplicate in 6-well plates at a density of 500 to 1000 cells per well for each treatment group. Cells were treated with the indicated concentrations of futibatinib and cultured for 3 weeks. The culture medium was changed and fresh futibatinib was added twice a week. The colonies were then fixed in 10% formalin and stained with 0.05% crystal violet in 25% methanol. The stained colonies in the wells were scanned, and total colony area and average colony size were quantitated using ImageJ v.1.48 software.

### Reverse phase protein arrays

RPPA were performed at the MD Anderson Cancer Center Functional Proteomics Core Facility. Frozen tumor pieces were cut into approximately 3 × 3 mm size fragments and placed in bead lysis tubes for protein extraction. We chose the RPPA results originated from rabbit and goat antibodies (230 antibodies) for PDXs because validated mouse antibodies have significant background in PDX samples due to the mouse stromal tissue.

### Bioinformatics analysis of *FGFR* aberrations in patients

To analyze *FGFR* mutations in breast cancer, we selected the MSK-IMPACT series^25^, the MBC Project (Provisional, February 2020)^24^, and the TCGA Project (PanCancer Atlas, Breast Invasive Carcinoma) datasets. The MSK-IMPACT, MBC Project, and TCGA datasets were downloaded from the cBioPortal for Cancer Genomics^22,23^. The MD Anderson database included breast cancer tumors that were analyzed either on FoundationOne (FoundationOne and FoundationOne CDx) or Oncomine (STGA-DNA 2018 and STGAv1) NGS platforms. The *FGFR* mutations were interpreted with protein annotations using the cBioPortal MutationMapper online tool. To analyze the expression of FGFR2 Y375C in various tissues, we searched the COSMIC database^26^.

#### Bioinformatics and statistical analysis

For the in vitro studies, comparisons between the growth metrics in 2 groups were performed using two-sample *t*-tests. All in vitro experiments were performed at least 3 times.

Tumor volume (TV; in mm^3^) was calculated as ([width]^3^ × length)/2. In vivo data are presented as means ± the standard errors of the means (SEMs). The change in TV from baseline was calculated as (TV dayX – TV day0)/TV day0. The tumor growth ratio to day 0 were calculated as a relative tumor volume on each day compared to treatment start day per animal. Tumor growth inhibition is defined as the ratio of mean tumor growth ratio of treatment group to mean of control group. ANOVA model was applied to estimate the tumor growth inhibition and the Delta methods were used to estimate their standard errors.

An event was defined as a doubling of the TV from the initial TV on day 0. EFS was defined as the time interval from initiation of study to the first event or the end of the study period for tumors that did not double in volume (censored cases). The time to event was determined using linear interpolation. The EFS T/C ratio was defined as the ratio of the median time to event of the treatment group and the median time to event of the respective control group. If the treatment group did not have a median time to event, then the EFS T/C ratio was defined as being greater than the ratio of the last day of the study for the treatment group divided by the median time to an event for the control group. Kaplan–Meier survival analyses were used for EFS and EFS T/C value calculations. The time to event was determined using linear interpolation. Comparison between control and treatment groups was performed using the log-rank test. Final day tumor volume comparisons between control and treatment groups were done using *t*-test. A *P*-value less than 0.05 was regarded as significant.

We aligned the T200 target-capture deep-sequencing data to human reference assembly hg19 using Burrows-Wheeler Aligner^65^ and removed duplicated reads using Picard^66^. All samples mean coverage > 300. We called single-nucleotide variants (SNV) and small indels using an in-house developed analysis pipeline^67^, which classified variants into three categories: somatic, germline, and loss of heterozygosity based on variant allele frequencies in the tumor and the matched normal tissues. We called copy-number alterations (CNA) using a previously published algorithm^68^, which reports gain or loss status of each exon. To understand the potential functional consequence of detected variants, we compared them with dbSNP, COSMIC^69^, and The Cancer Genome Atlas (TCGA) databases, and annotated them using VEP^70^, Annovar^71^, CanDrA^72^ and other programs. For Paired T200 samples, variants that were only detected by ClinSek^73^ and had an allele frequency less than 10% were filtered. Unpaired T200 used a pooled normal sample to use the paired T200 pipeline. In addition, recurrent variants of unpaired samples were filtered out and then we filtered out more variants based on COSMIC_EXACT_MATCH, 1000 genome MAF and ESP6500 MAF. A deletion was defined as a loss of copy number less than or equal to 0.6. An amplification was defined as a gain of copy number greater than or equal to 5.

The RNA-seq read counts were normalized with “DESeq2”^74^. Boxplot, unsupervised hierarchical clustering, principal component analysis (PCA), and heatmap for most variant genes were used for quality assessment. The read counts were fitted with a negative binomial generalized linear model and tested with Wald statistics. Variance stabilizing transformation (VST) was used in visualization, clustering, and PCA. VST is the transformed data on the log2 scale which has been normalized with respect to library size or other normalization factors. The differentially expressed genes (DEG) were identified with a specified FDR. Twenty-one PDX models had RNAseq data^16^. Eighteen models had paired patient and PDX RNAseq data. These samples were generated in 2 batches. After the removing the batch effect, gene expression was shown in log2-transformed reads per kilobase million (RPKM). The Pearson correlation coefficient (*r*) was calculated using log2-normalized counts.

### Supplementary Information

Supplementary Information.

## Data Availability

The datasets MSK-IMPACT Clinical Sequencing Cohort (MSK, Nat Med 2017) (https://www.cbioportal.org/study/summary?id=msk_impact_2017), The Metastatic Breast Cancer Project (Provisional, December 2021) (https://www.cbioportal.org/study/summary?id=brca_mbcproject_2022), and TCGA PanCancer Atlas, Breast Invasive Carcinoma (https://www.cbioportal.org/study/summary?id=brca_tcga_pan_can_atlas_2018) analyzed during the current study are available in the cBioPortal for Cancer Genomics repository. FGFR2 Y375C (COSMIC gene: FGFR2 (COS95033) mutation data was analyzed using COSMIC database (https://cancer.sanger.ac.uk/cosmic/gene/analysis?ln=FGFR2). The institutional dataset analyzed during the current study is not publicly available as it contains individually identifiable information. A redacted dataset is available from the corresponding author on reasonable request.
